# Mutated DNA Damage Repair Pathways Are Prognostic and Chemosensitivity Markers for Resected Colorectal Cancer Liver Metastases

**DOI:** 10.3389/fonc.2021.643375

**Published:** 2021-03-31

**Authors:** Kun Wang, Ming Liu, Hong-Wei Wang, Ke-Min Jin, Xiao-Luan Yan, Quan Bao, Da Xu, Li-Jun Wang, Wei Liu, Yan-Yan Wang, Juan Li, Li-Juan Liu, Xiao-Yu Zhang, Chun-He Yang, Ge Jin, Bao-Cai Xing

**Affiliations:** ^1^ Key Laboratory of Carcinogenesis and Translational Research (Ministry of Education/Beijing), Hepatopancreatobiliary Surgery Department I, Peking University Cancer Hospital, Beijing, China; ^2^ GloriousMed Clinical Laboratory (Shanghai) Co., Ltd., Shanghai, China

**Keywords:** colorectal cancer liver metastasis, DNA damage repair, next-generation sequencing, prognosis, chemo-sensitivity

## Abstract

Deficiency of the DNA damage repair (DDR) signaling pathways is potentially responsible for genetic instability and oncogenesis in tumors, including colorectal cancer. However, the correlations of mutated DDR signaling pathways to the prognosis of colorectal cancer liver metastasis (CRLM) after resection and other clinical applications have not been fully investigated. Here, to test the potential correlation of mutated DDR pathways with survival and pre-operative chemotherapy responses, tumor tissues from 146 patients with CRLM were collected for next-generation sequencing with a 620-gene panel, including 68 genes in 7 DDR pathways, and clinical data were collected accordingly. The analyses revealed that 137 of 146 (93.8%) patients had at least one mutation in the DDR pathways. Mutations in BER, FA, HRR and MMR pathways were significantly correlated with worse overall survival than the wild-types (P < 0.05), and co-mutated DDR pathways showed even more significant correlations (P < 0.01). The number of mutated DDR pathways was also proved an independent stratifying factor of overall survival by Cox multivariable analysis with other clinical factors and biomarkers (hazard ratio = 9.14; 95% confidence interval, 1.21–68.9; P = 0.032). Additionally, mutated FA and MMR pathways were positively and negatively correlated with the response of oxaliplatin-based pre-operative chemotherapy (P = 0.0095 and 0.048, respectively). Mutated DDR signaling pathways can predict pre-operative chemotherapy response and post-operative survival in CRLM patients.

## Introduction

Colorectal cancer (CRC) is the third most common cancer worldwide and the second leading cause of cancer-related deaths (1). Approximately 50% of patients diagnosed with colorectal cancer will develop liver metastases during their disease. The liver is the most common site of dissemination and causes two thirds of death. Surgical resection of colorectal liver metastases (CRLM) remains the only potentially curative therapy, with 5-year survival rates exceeding 50% in many series. Unfortunately, of patients who undergo liver resection, 50% to 75% will develop disease recurrence within 2 years after resection (2, 3). Therefore, accurate prognostic markers are needed for risk stratification and optimization of patient selection for hepatic resection. However, the prognostic landscape for predicting long-term outcomes in patients undergoing CRLM resection is changing (4–8). In the past 20 years, clinicopathological factors had been gradually established and applied. Recent studies have focused on molecular alterations in CRLM for risk stratification. Specifically, some tumor-related genomic alterations, such as RAS/RAF, are necessary to guide patient selection not only for target therapies but also for hepatic resection and related treatments to achieve the best clinical benefit (5–8). As our understanding, the molecular and genetic determinants of metastatic colorectal cancer’s outcomes continue to expand, the importance of these molecular biomarkers in the personalized management of CRLM will only continue to increase.

Since next-generation sequencing (NGS) technology has been widely applied, it is now possible to evaluate a large number of genes and samples extensively and rapidly for prognostic and therapeutic response potentials. Previously integrative genomics analysis has revealed that colorectal cancer usually starts from benign lesions, and accumulation of DNA damage leads to cancer progression to more metastatic and invasive forms (9–11). Seven functional signaling pathways are involved in DNA damage repair (DDR): homologous recombination (HRR), mismatch repair (MMR), base excision repair (BER), nucleotide excision repair (NER), nonhomologous end-joining (NHEJ), checkpoint factors (CPF), and Fanconi anemia (FA) (10, 11), with defective MMR being established as an essential factor in colorectal cancer pathogenesis, treatment, and outcome (12). However, the mutational landscape of DDR pathways and their clinical implications of pre-operative chemotherapy sensitivity and post-operative prognosis has not yet been systematically explored in CRLM. Therefore, in the present study, we aimed to investigate the DDR mutational profile and its impacts on the outcome of patients undergoing liver resection for CRLM.

## Materials and Methods

### Patients and Sample Collection

One hundred forty-six patients who underwent liver resection for CRLM with curative intent at The Beijing Cancer Hospital between January 2015 and February 2017 were included in this study. formalin-fixed paraffin-embedded (FFPE) tissue samples from metastatic liver lesions were collected. Peripheral blood or adjacent healthy tissues were collected from each patient as controls for genomic profiling. Hematoxylin and eosin-stained sections from each tissue sample were subjected to independent pathological reviews to confirm that the tumor specimen was histologically consistent with metastatic tumors (>20% tumor cells) and that the adjacent tissue specimen contained no tumor cells. Demographic and clinicopathologic characteristics and outcomes were collected. This study was conducted in accordance with the Declaration of Helsinki. All patients were acknowledged of the study with informed consent and had granted permission to being included. For survival analyses, overall survival (OS) was examined from liver resection to date of death. Disease-free survival (DFS) was calculated from the date of liver resection until tumor recurrence.

### Next-Generation Sequencing

DNA from FFPE tumor tissue samples and patient-matched adjacent healthy tissues or normal blood samples were extracted using the DNA Extraction Kit (QIAamp DNA FFPE Tissue Kit or CWBio Blood Genomic DNA Mini Kit [CW2087M]). Then the DNA was sheared into 150 to 200 bp fragments with Bioruptor^®^Pico Instrument (Diagenode, Seraing, Belgium). Fragmented DNA libraries were constructed by The KAPA Hyper Prep Kit (KAPA Biosystems, Wilmington, MA, USA) following manufacturer’s instruction. DNA libraries were captured with a designed panel of 620 key cancer-related genes (GloriousMed, Shanghai, China). The captured samples were subjected to Illumina HiSeq X-Ten for sequencing. Sequencing adapters were trimmed by Trimmomatic from the raw data (13). Duplicated reads were removed by Picard (http://broadinstitute.github.io/picard/). Mapped reads were also realigned to the genome by Genome Analysis Tool Kit 3.7 (14). Somatic mutations were called by Mutect2 and GATK’s HaplotypeCaller (3.7) with a paired workflow and GATK (3.7) respectively (14). Variants were then annotated by ANNOVAR (v-xxx) and self-development code (15). An in-house script was used to verify the human identity concordance of paired samples, and known germline alternations in dbSNP were excluded. Mutations were then filtered with the threshold of 2% in allele frequencies and >8 mutant reads for hotspot mutations, and 5% in allele frequencies, >10 mutant reads for non-hotspot mutations (16).

### Statistical Analysis

For comparison of genomic alterations, targeted sequencing data of 195 samples from stage IV liver biopsy and metastasectomy was selected from an 1134 metastatic colorectal tumor/normal pairs database downloaded from cBioPortal (17–19). Sequencing results were trimmed to fit the Memorial Sloan Kettering (MSK)-IMPACT 341 gene assay for comparison of mutation consistency between the two datasets using two-sided Fisher’s exact test. Kaplan–Meier survival curves were generated and compared using the log-rank test. Multivariable survival models were computed using Cox proportional hazards regression. Correlation of DDR mutations with pre-operative chemosensitivity was analyzed by Fisher’s exact test. Statistical significance thresholds were set to a two-tailed 0.05 value. R software (version 3.6.1) was used for statistical analyses.

## Results

### Study Populations

A total of 146 patients with CRLMs underwent hepatectomy between January 4, 2015, and February 24, 2017, in the Hepatopancreatobiliary Surgery Department I at the Beijing Cancer Hospital and Institute (Beijing, China). 29 (19.8%) of patients went directly to surgery, 117 (80.1%) had pre-operative chemotherapy (**Supplementary Figure 1**). Demographic and clinicopathologic characteristics of all patients were summarized in **Table 1**. All patients provided written informed consent, and the ethical review board committee approved the study of the Beijing Cancer Hospital and Institute. Information on specific regimens and efficacy evaluation of pre-operative chemotherapy with or without target agents were collected in 112 of 146 patients. According to the World Health Organization criteria, the response to chemotherapy was classified, which agrees with the Response Evaluation Criteria in Solid Tumors (RECIST). Treatment response was evaluated to assess the possibility of through surgery in a multidisciplinary discussion. Numerous studies have demonstrated that a tumor’s response to pre-operative chemotherapy (TRC) is an important predictive factor for evaluating long term survival in patients with CRLMs (17–20). The good TRC group (response to pre-operative chemotherapy) included 66 patients with a complete or partial response and those with a response within a stable disease status (a reduction in the sum of tumor diameters of <30%), while the bad TRC group comprised of 41 patients with progressive disease or progression within a stable disease status (an increase in the sum of the diameters of the target lesion of <20%). The median duration of follow-up was 39.5 months (range, 7–64 months). During the follow-up period, 73 (50.0%) patients died and 108 (74.0%) patients experienced recurrence.

**Table 1:** Clinical characteristics and pre-operative plans of the study population.

Characteristics	Number of concerns
**Gender**	
Male	94
Female	52
**Age, median (range)**	58 (37–80)
**Primary site**	
Right colon	20
Transverse colon (counted right)	4
Left colon	48
Sigmoid colon (counted left)	14
Rectum	60
**Liver metastases occurrence**	
Synchronous	93
Metachronous	53
**Direct surgery**	29
**Pre-hepatectomy CEA level, median (IQR), ng/mL**	7.01 (0.613 – 651.5)
**Number of metastases, median (range)**	2 (1–25)
**Size of largest liver metastasis**	
<5	132
≥5	14
**Resection margin**	
R0	115
R1	31
**Pre-operative therapy (regimen specified)**	112
Oxaliplatin-based	79
Irinotecan-based	32
Other	1

### Mutation Profile and Survival Analyses for Key Genes in Our Cohort

**Figure 1:**
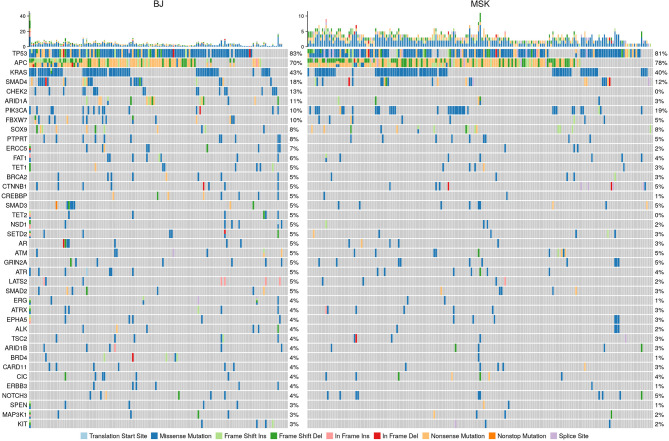
Mutation spectrum comparison of our cohort with the 195 CRLM samples of the MSK data set (Yaeger et al. 2018). Our sequencing results were trimmed according to the standards and gene panel of the other dataset to maintain comparability. The distribution of genes and mutation were consistent between the two datasets, especially for the essential genes with high occurrences, such as TP53, KRAS, APC and PIK3CA.

The mutation profile of our data and the mutation profile comparison with the MSK CRLM dataset were shown in **Figure 1**. The gene distributions were similar in important oncogenic genes between the MSK CRLM and our dataset. The most frequently mutated genes in our cohort were *TP53* (82.9%), *APC* (69.9%), *KRAS* (43.2%), *SMAD4* (17.8%), *CHEK2* (13.0%), *ARID1A* (11.0%), *PIK3CA* (10.3%), *FBXW7* (10.3%), *AMER1* (10.3%), *BRCA2* (5.5%), *CTNNB1* (5.5%), etc.

### The DDR-Related Pathway Mutation

**Figure 2:**
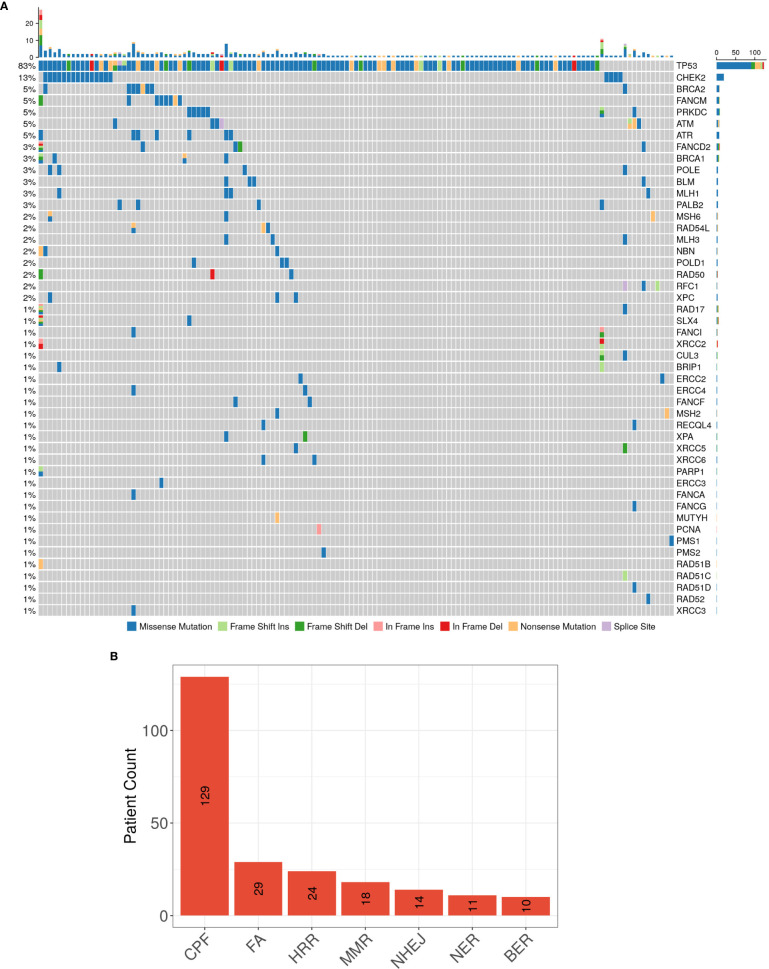
**(A)** The spectrum of DDR genes with detected somatic mutations, and **(B)** the ranking of patients carrying mutations in the 7 DDR signaling pathways in our CRLM cohort NGS results.

Despite the consistency in genes with high mutation occurrences, the DDR-related genes, such as *CHEK2* and *ARID1A*, appear to be significantly more frequently mutated in our population than that in the MSK CRLM population (**Supplementary Table S1**). To depict the profile of DDR pathway mutations in our cohort, we referred to a category including 68 genes in 7 DDR pathways: MMR, BER, CPF, FA, HRR, NER, and NHEJ, according to Wang et al. (20) (**Supplementary Table S2** **)**. 137 of 146 (93.8%) patients had at least one mutation in genes of the covered DDR signaling pathways. The most frequently mutated individual DDR gene was *TP53* (82.9%), followed by *CHEK2* (13.0%), *BRCA2* (5.5%), *FANCM* (5.5%), *PRKDC* (4.8%), *ATM* (4.8%), *ATR* (4.8%), *FANCD2* (3.4%), *BRCA1* (2.7%), *POLE* (2.7%), *BLM* (2.7%), *MLH1* (2.7%) and *POLD1* (2.0%), *etc*. (**Figure 2A**). The signal pathway with the most mutations detected was the CPF signal pathway, in which 88.4% (129/146) of patients carried mutations. This high proportion might be caused by the high frequency of mutations in the *TP53* gene belonging to this pathway. The ranking of the mutation ratios of other DDR pathways were shown in **Figure 2B**.

### Mutated DDR Pathways Predicted Worse OS After CRLM Resection

**Figure 3:**
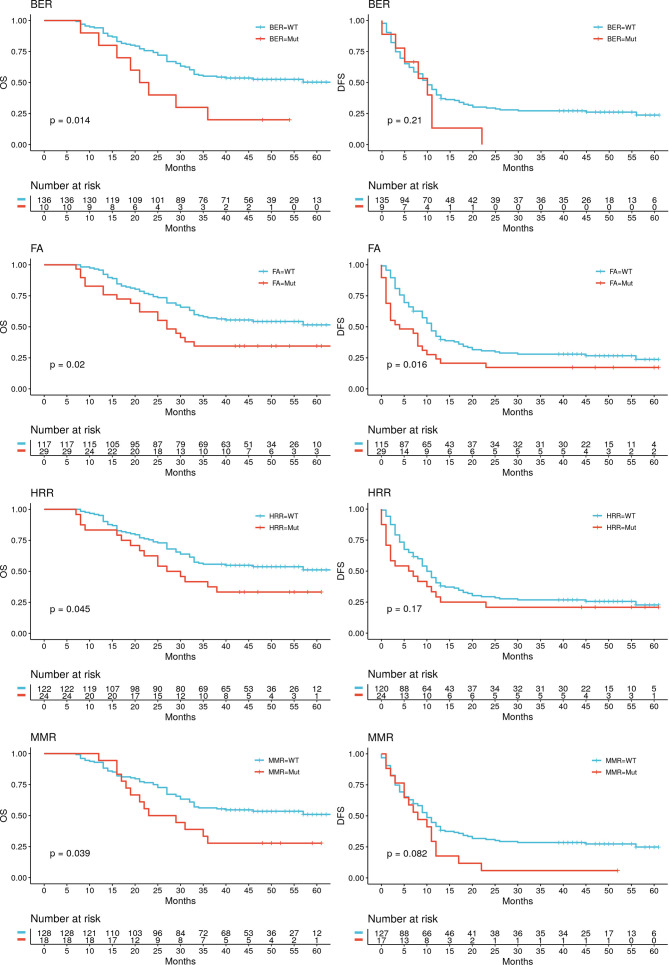
Kaplan-Meier curves of survival differences in patients with or without mutations in certain DDR pathways. OS in four of the DDR pathways showed significant differences between the mutated and wildtype patients: BER, FA, HRR and MMR. The patients carrying mutations in these four pathways are statistically having shorter OS and thus poorer prognosis than the wildtype ones. DFS in patients with or without mutations in the above pathways showed no significant difference, except for the FA pathway. The curves of other pathways without any significance in results are attached in **Supplementary Figure 3**.

As most DDR genes have not yet been well studied, we defined mutations in DDR pathways as any mutations in the corresponding pathways, including missense, nonsense, insertion, deletion, splice, and multi-hit mutations. A significant difference of OS was found between the patients with or without any DDR pathway mutation (P = 0.039), but the disparity of sample sizes with a wild-type subgroup of only nine patients might have compromised the statistic power. Therefore, further evaluations were conducted separately in the seven specific DDR pathways. The correlations between mutations and OS after resection of CRLM are shown in **Figure 3**, that mutations in BER, FA, HRR and MMR pathways were significantly associated with shorter OS (mOS: BER mutation [mut] vs. wild-type [wt], 22 months vs. not reached [NR], P = 0.014; FA mut vs. wt, 27 months vs. NR, P = 0.021; HRR mut vs. wt, 28.5 months vs. NR, P = 0.047; MMR mut vs. wt, 26 months vs. NR, P = 0.038). DFS also distinguishably differed between mutated and wild-type subgroups of the above pathways, but the difference appeared significant only concerning the FA pathway (mDFS FA mut vs. wt, 4 vs. 11 months, P = 0.016). Additionally, no significant difference in either OS or DFS outcomes was found in patients with CPF, NER and NHEJ pathway alterations and the wild-types (**Supplementary Figure 2**).

### DDR Co-Mutations and Quantity of Mutated DDR Pathways Predicted Better Stratification of Post-Operative Survival in CRLM Patients

**Figure 4:**
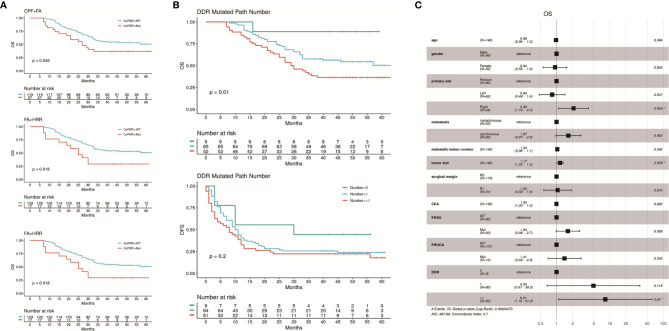
**(A)** Kaplan-Meier curves of OS and DFS showing differences between subgroups with and without specific co-mutations of two DDR pathways. The subgroups carrying co-mutations in CPF + FA or FA + HRR pathways had significantly worse OS, and the FA + HRR subgroup also had significantly worse DFS. The results with insignificant correlations were shown in **Supplementary Table 3**. **(B)** the patients carrying mutations in more than one DDR pathway had the worst OS comparing with those with 1 or 0 mutated DDR pathway mutations. The DFS of these three subgroups were also distinguishable, but with less significance. **(C)** Result of multivariable Cox proportional hazards regression analysis of OS, including the number of mutated DDR pathways, as well as clinical factors previously reported independently associated with CRC prognosis. Carrying more than one mutated DDR pathways maintained significant negative correlation with OS (HR = 9.14, 95% CI: 1.21 – 68.9). The analysis of DFS showed no significance, and result was attached in **Supplementary Figure 4**.

To investigate whether co-mutations of specific DDR pathways could have combined and more significant effect than single DDR pathway mutations on the patients’ survival, we compared the survival data of subgroups with and without co-mutations in every two of the seven DDR pathways. Co-mutations in the pathways of CPF + FA and FA + HRR, in which the difference showed particular significance between the mutated and the wild-types (mOS: CPF + FA co-mut vs wt, 27 months vs NR, P = 0.045; FA + HRR co-mut vs wt, 25 months vs NR, P = 0.018; mDFS FA + HRR co-mut vs wt, 2 vs 11 months, P = 0.0058), and the lower P value also demonstrated more significance in stratifying OS or DFS than the two single pathways considered independently (**Figure 4A** **;** **Supplementary Table 3**).

Additional analyses on the correlation between numbers of mutated DDR signaling pathways with survival also revealed that subgroups with higher amount of mutated DDR signaling pathways had significantly worse OS (P = 0.01). The patients carrying mutations in genes in more than one DDR pathway had a mOS of 29.5 months, while the ones with 1 or 0 mutated DDR pathway showed mOS not yet reached. The DFS of these three subgroups were also distinguishable, but with less significance (median DFS [mDFS]: 8.0 vs 10.5 vs 30.0 months, respectively, P = 0.2; **Figure 4B**).

### Multivariable Hazard Ratio Revealed the Correlation of DDR Pathway Mutations and Other Biomarkers in This Cohort

Clinical factors previously reported independently associated with CRC prognosis were entered in a Cox proportional hazards regression model: age, gender, primary tumor sites, metastatic synchronicity, metastatic lesion number, metastatic tumor size, surgical margin, pre-operative carcinoembryonic antigen (CEA), together with the number of mutated DDR signaling pathways. The known prognostic biomarkers, *KRAS* and *PIK3CA* (21–25), which were consistently proved significantly correlated with worse OS in our study population (**Supplementary Figure 3**), were also taken into analysis. Carrying more than one mutated DDR pathways maintained significant negative correlation with OS (HR, 9.14; 95% CI, 1.21–68.9), but not with DFS. Primary site in right colon (HR, 2.325; 95% CI, 1.178–4.588), larger tumor size (HR, 1.17; 95% CI, 1.02–1.3) and *KRAS* mutation (HR, 1.73; 95% CI, 1.03–2.8) were also significantly correlated with OS. No other factor was found significantly associated with either OS or DFS in the Cox regression model (**Figure 4C**; **Supplementary Figure 4**).

### The FA and MMR Signaling Pathways Showed Correlations With Efficacy of Oxaliplatin-Based Pre-Operative Therapies

**Figure 5:**
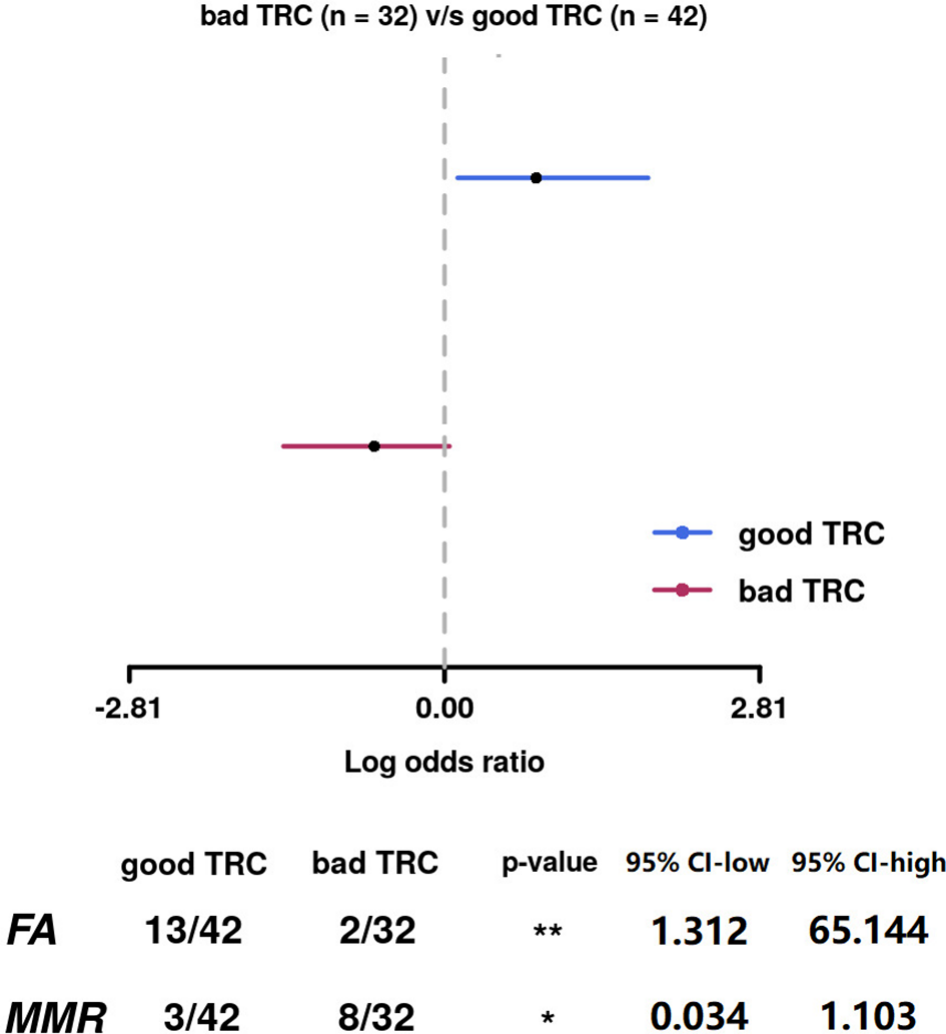
The efficacy of oxaliplatin-based treatment was positively correlated with FA pathway mutations, while negatively correlated with MMR pathway mutations. The correlations were both significant.

We analyzed whether the mutations in each DDR pathway were related to the efficacy of oxaliplatin- and irinotecan-based pre-operative treatments. The subgroup of patients in the irinotecan subgroup is too small (32/146) and thus the analyses showed low statistical power. In the 79 patients experienced oxaliplatin-based pre-operative treatment, the efficacy of oxaliplatin-based treatment was positively correlated with FA pathway mutations (good TRC% of FA-mutated group: 31.0%, of FA-wild-type group: 6.3%), while negatively correlated with MMR pathway mutations (good TRC% of MMR-mutated group: 7.1%, of MMR-wild-type group: 25.0%). The correlations were both significant (**Figure 5**).

## Discussion

Regular functions of DDR are essential to regular replication and metabolism for cells. Mutations that may influence the functions of DDR signaling pathways would cause genomic instability and thus the accumulation of mutations, DNA base mismatches, and chromosomal abnormalities. Although there are already several studies about the clinical significance of specific DDR genes, such as *BRCA1/2*, *POLE*, *POLD1*, and *MLH1* (26–31), studies about correlations of DDR pathway somatic mutations with the prognosis of CRC that consider the DDR pathways as a whole are still lacking. Herein, we investigated the mutational distribution and clinical significance of DDR signaling pathways in 146 patients with CRLM after resection. We demonstrated that the existence and quantity of mutated DDR pathways might correlate with survival after liver resection and pre-operative chemotherapy response for CRLM patients.

Single gene biomarkers of CRC, such as *TP53*, *APC*, *KRAS*, and *PIK3CA*, have already been well-recognized of their high populational mutation occurrences, as well as their significant correlations with CRC prognosis (32–36). Previous study on Chinese CRC patients with brain metastases also reveals modified DDR gene signature, homologous recombination deficiency and mismatch repair deficiency in brain metastases than the primary lesions (37). Therefore, considering the mutational status of DDR pathways, which is possibly unique to metastatic CRC patients, may help provide a more comprehensive reference for treatment and surveillance.

Different DNA damage forms evoke responses by different repair-related signaling pathways (38, 39). Alterations in DDR pathways could hinder the DNA repair capacity, inducing those that confer genetic and chromosomal instability, and each of the DDR pathways possesses a specific function and collaborate in DNA repairment. The BER pathway is mainly responsible for DNA single-strand breaks, which are the most common type of DNA damage (40–42). The HRR pathway answers DNA double-strand breaks (39, 42), and the FA pathway aims for DNA inter-strand crosslinks (39, 43, 44). Although the loss of function in one or more DDR signaling pathways can, to some extent, be compensated by other pathways (44), due to the generally considered mutually exclusive and distinct functions of each, the outcomes could potentially accumulate the influence on survival, causing significant damage. Mice embryo studies have shown synthetic lethality of HRR and NHEJ pathways (45, 46). Defective variants in *POLD1* and *POLE*, essential genes in the BER pathway, are related to significantly higher mutational burden and malignancy through BER’s correlation to the MMR pathway (47). Co-mutations in the MMR and HRR pathways may also be related to hypermutated CRC with worse survival, via interruption of DNA binding and replication (48). Our study reveals that beyond each single DDR pathway mutations, the co-mutations and the number of mutated DDR pathways are also significantly related to post-operative survival, and the correlations were independent of other clinical traits. Even though the sparsity of patients with mutations possibly influenced the statistical power in each of the overlaps, these results indicated that not only mutations in separate DDR pathways are prognostic-related in our cohort, but the effect could also act additively with possibly better stratification power when considered together.

Beyond mutations in DDR pathways, multivariate Cox analysis also indicates that other known prognostic biomarkers, such as right colon-primary, larger tumor size and *KRAS* mutations, could act accumulatively with DDR pathway mutations on influencing the OS, enlightening further clinical explorations of for stratification of risks of CRLM patients. According to previous studies, DDR mutations are more frequently detected in right colon-primary sites than left colon-primary cases (49), indicating probable developmental differences. Molecular analysis has shown that *POLE* damaging variants may influence the oncogenesis through the RAS/RAF signaling pathway (50). *KRAS* activating mutations also present augmentation to the expression of HRR signaling pathway in *in vitro* study (51). However, the mechanistic details and specific molecular collaborations concerning clinical application may still require further researches.

The effects of platinum-based chemotherapy on DNA are mainly intra-strand crosslink and inter-strand crosslink (46, 52), which are primarily repaired by the FA/BRCA pathway. The normal or overexpression of the FA pathway has been discovered to be one of the mechanisms of platinum resistance in various cancers, including ovarian cancer. Multiple studies on ovarian cancer cell lines have shown that FA-deficiency induced by FA pathway inhibitors, such as bortezomib and curcumin, can sensitize the cell line to cisplatin treatment (39, 43, 52, 53). Other studies also showed that the MMR pathway’s normal function is necessary for detecting and repairing DNA damages caused by platinum-based chemotherapy. With MMR defective, tumor cells can resist DNA damage caused by platinum and continue to proliferate. MMR deficiency has been considered as a related pathway of cisplatin resistance in many studies. Ovarian adenocarcinoma cell line research has revealed that loss of hMLH1 or hMSH2 can lead to an approximately two-fold increase in cisplatin, and a 1.3-fold increase in carboplatin resistance (53, 54). Studies on ovarian cancer cell lines have also shown that the MMR pathway’s inactivation can reduce the sensitivity to cisplatin and carboplatin, yet has no significant effect on oxaliplatin (55). With no confirmed results concerning DDR pathway mutations and the efficacy of platinum-based therapies in CRLM, our results were mostly consistent with other cancers’ existing studies, while also called on more specific and CRLM-related studies. Moreover, instead of focusing on merely the essential genes, we considered FA and MMR pathways as a whole, which may have better coverage for clinical application. However, our study has inevitable limitations that the tumor tissues are sampled from resections after the neo-adjuvant or conversion chemotherapy, and the number of patients in each subgroup is small. This may have caused the controversy that patients with FA pathway mutations present better TRC to oxaliplatin-based pre-operative treatment but worse OS than the FA wildtypes. As shown in **Supplementary Table 4**, among all patients carrying FA pathway mutations, the subgroup showing good oxaliplatin TRC appeared to have more metastatic lesions and synchronous metastases. Both factors have been reported to correlate significantly independent of treatment with shorter OS in mCRC (56, 57). On the other hand, the higher pre-operative CEA levels and more patients undergoing direct surgeries presented in the subgroup without good oxaliplatin TRC are also negatively correlated with the survival of mCRC (58–61). Therefore, when all patients carrying FA pathway mutations were considered as a whole in survival analyzes, the positive effect of chemotherapeutic response may have been compromised by other negative factors listed above, especially in small populations as in this study. Further verifications would be needed to avoid the above compromising factors.

In conclusion, mutations in DDR signaling pathways may predict worse post-operative survival in our CRLM patients. Nevertheless, studies with larger sample sizes and better coverage of DDR-related genes are pivotal for further verifications. Clinical explorations are also ongoing to use the poly (ADP-ribose) polymerase (PARP) inhibitors in colorectal cancer patients carrying DDR inactivation and have benefited from previous platinum chemotherapy (62, 63). These findings may be useful for clinical decisions in patients with tumor characteristics associated with poor prognosis and risk stratification of patients in future clinical studies.

## Data Availability Statement

The raw data supporting the conclusions of this article will be made available by the authors, without undue reservation.

## Ethics Statement

The studies involving human participants were reviewed and approved by Ethics Committee of Peking University Cancer Hospital. The patients/participants provided their written informed consent to participate in this study.

## Author Contributions

B-CX and KW conceived and designed the study. ML, H-WW, K-MJ, QB, DX, and L-JW contributed to the clinical samples and informed consents collection. JL and L-JL provided clinical information. C-HY and X-LY conducted the bioinformatics analyses. X-YZ wrote the first draft of the manuscript. B-CX, KW, ML, and GJ reviewed and edited the manuscript. All authors contributed to the article and approved the submitted version.

## Funding

This study was supported by the grant (no. 31971192) from the National Nature Science Foundation of China.

## Conflict of Interest

X-YZ, C-HY, and GJ are employed by the company GloriousMed.

The remaining authors declare that the research was conducted in the absence of any commercial or financial relationships that could be construed as a potential conflict of interest.
