# Supplementary figures and images for: Mutated DNA Damage Repair Pathways Are Prognostic and Chemosensitivity Markers for Resected Colorectal Cancer Liver Metastases
Source: Front Oncol. 2021 Mar 31;11:643375. doi: 10.3389/fonc.2021.643375 (PMC8045762; doi:10.3389/fonc.2021.643375)

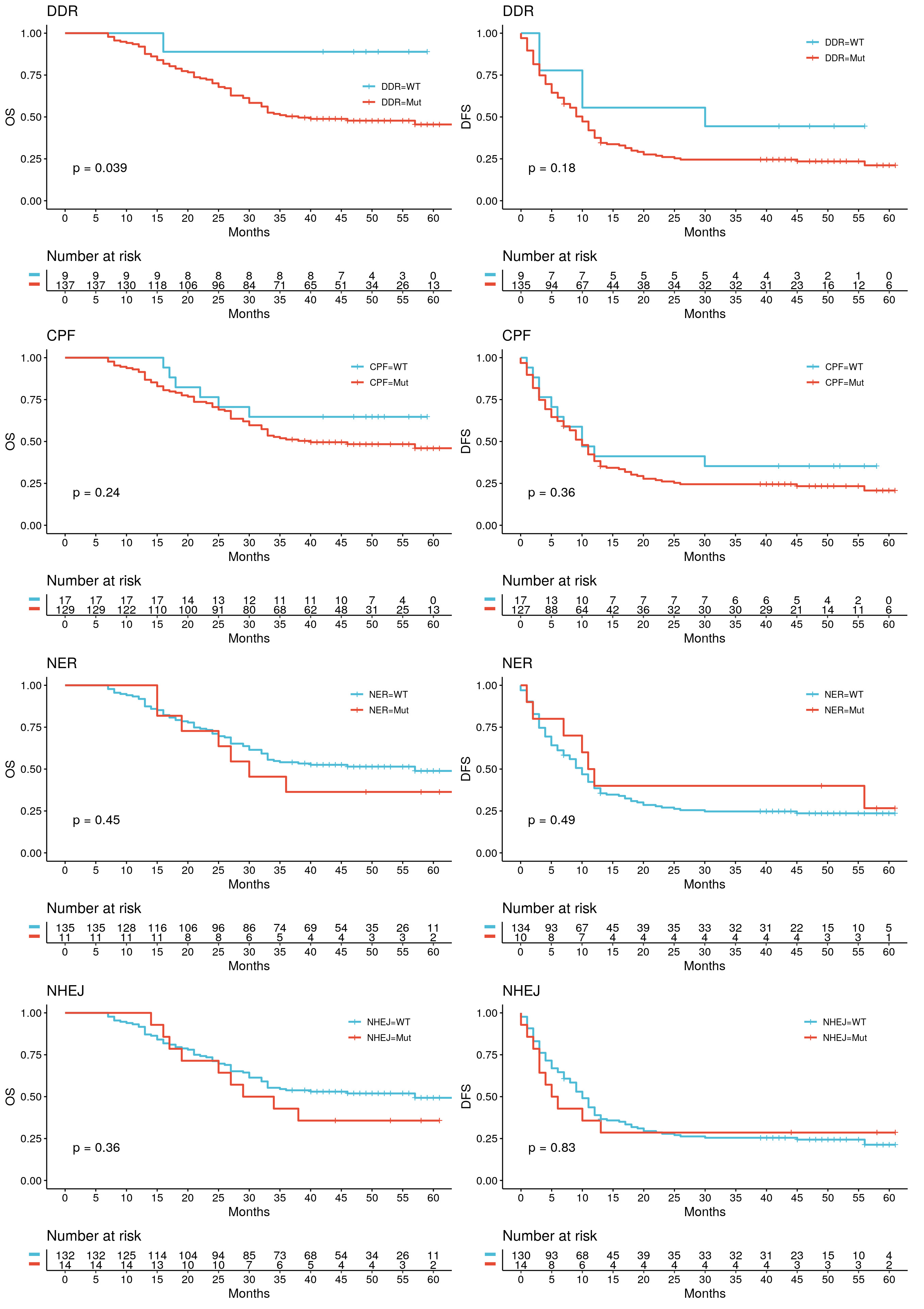

Supplement: Supplementary file 1 [file Image_1.tif]

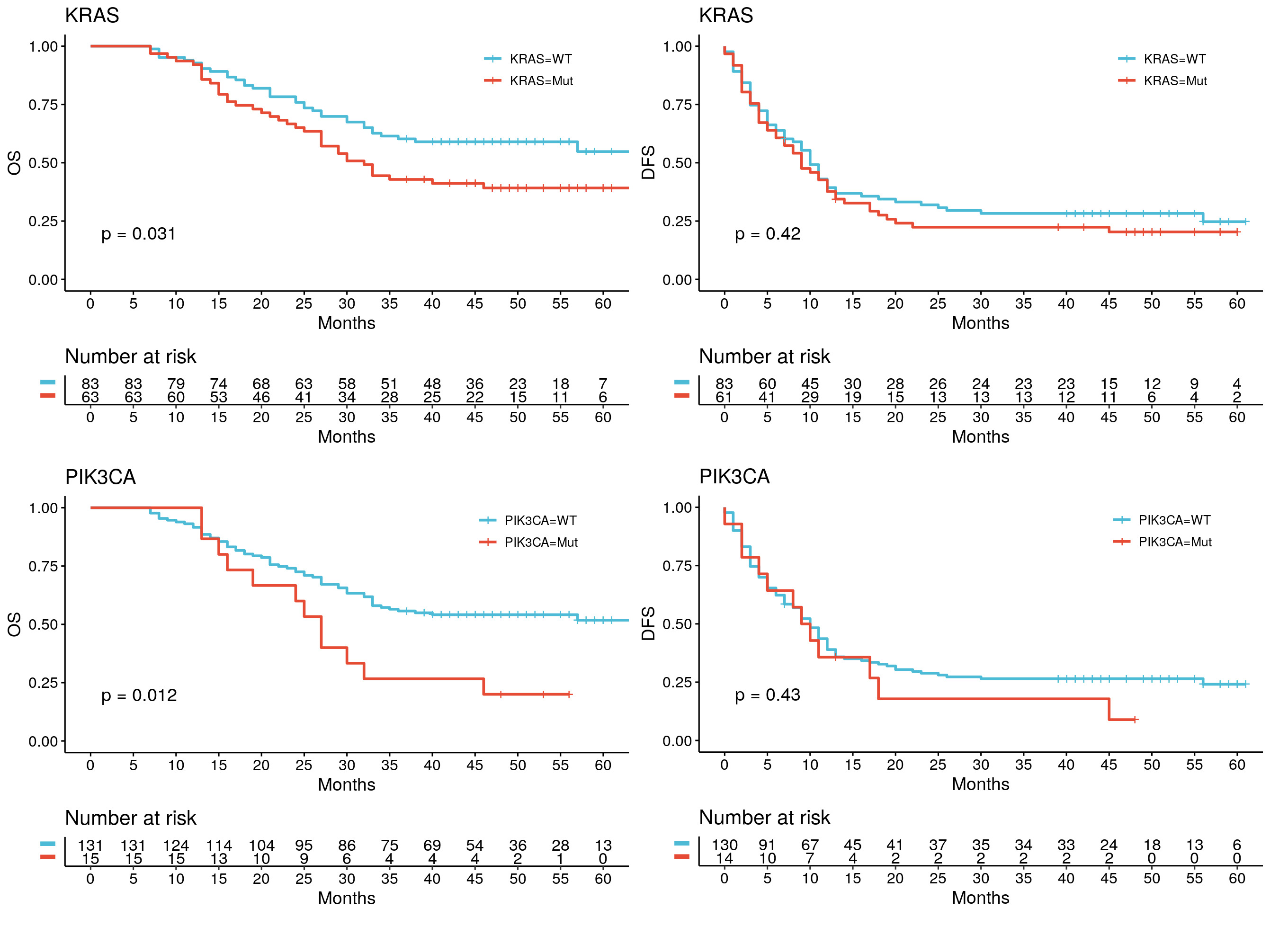

Supplement: Supplementary file 2 [file Image_2.tif]

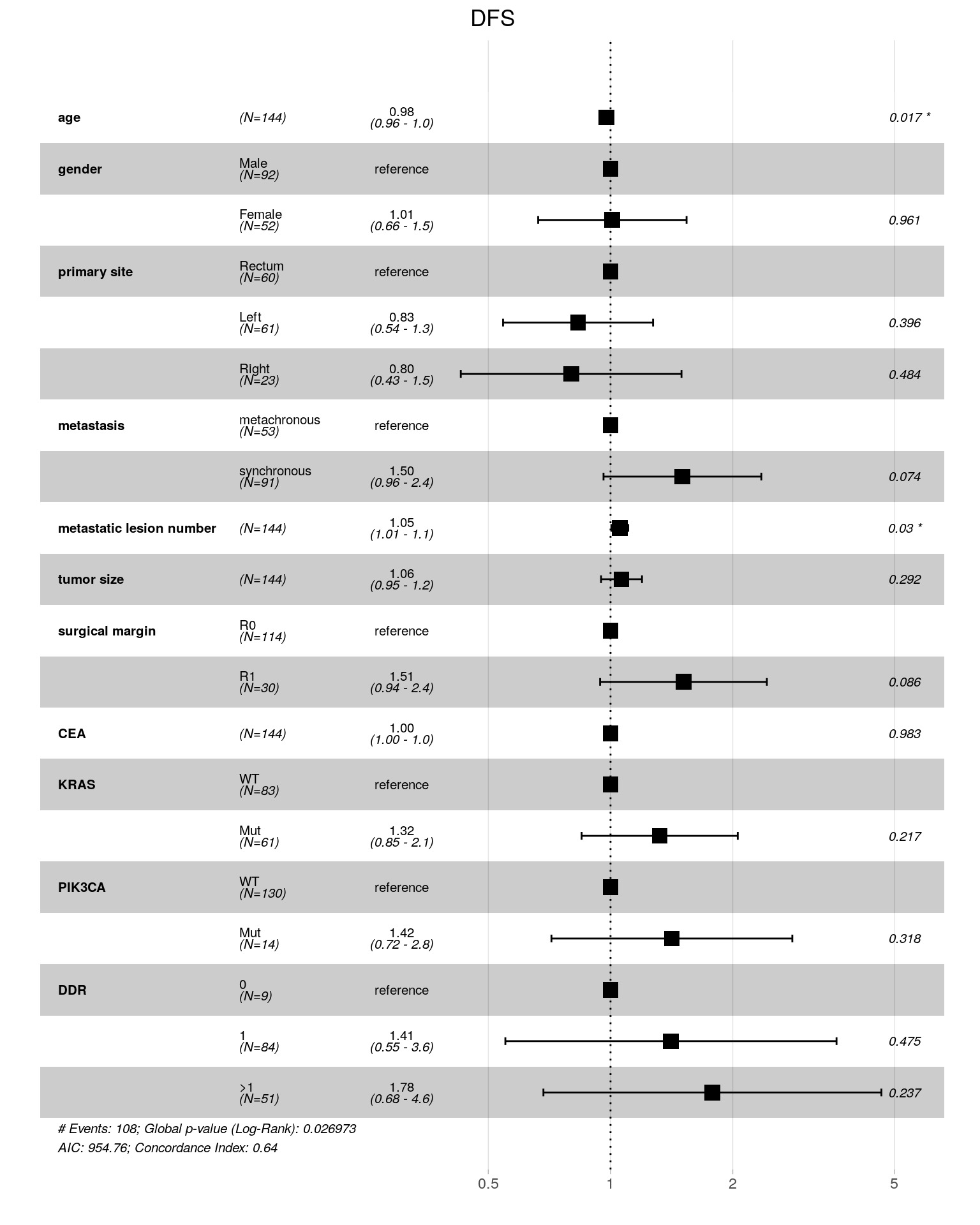

Supplement: Supplementary file 3 [file Image_3.tif]

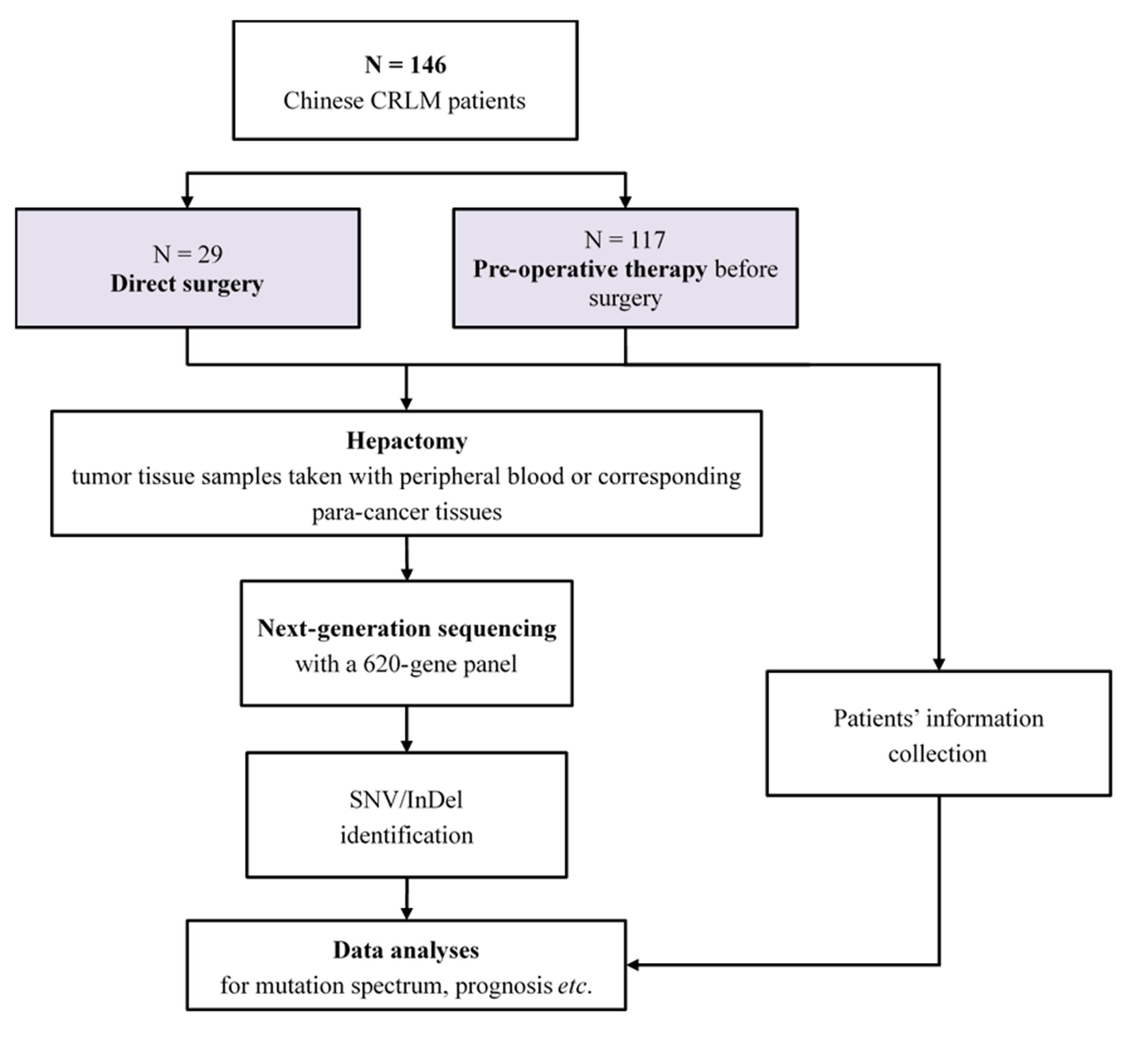

Supplement: Supplementary file 4 [file Image_4.tif]
